# The Man-Fish of Tennessee

**Published:** 1878-12

**Authors:** 


### EXCERPTA.

THE MAN-FISH OF TENNESSEE.—A short time since the Tennessee and Kentucky newspapers contained a startling account of a wild man lately captured, with great difficulty, in the Cumberland Mountains. He was six feet ten inches high, extraordinarily fleet of foot, and excessively savage. He fed chiefly on raw fish, which he captured without artificial aid. He spent much of his time in the water, and after being captured he had to be frequently bathed. He was covered with shining scales, like those of a fish. His hands and feet were webbed like the feet of water-fowls—so the newspaper accounts, with many embellishments, ran. It is scarcely necessary to say that much of this story was only showman's talk, uttered to attract the attention of the curious and credulous public.

The physicians of Louisville were invited to visit the monster upon his arrival in the city prior to his general exhibition. Among others I visited the merman; but before seeing the case I had diagnosed it as one of *icthyosis*, and a single glance was sufficient to verify the correctness of my conjecture. The man-fish presents a most magnificent example of the form of icthyosis, or fish-skin disease, called icthyosis serpentina, or serpent-skin; and his general effect is more that of a serpent than of a fish. But upon different parts of his body may be found nearly all the varieties of icthyosis. The resemblance of this man's skin to the shed skin of a boa-constrictor, lately brought me by a friend from the zoological garden in London, is almost perfect. About his joints the skin is loose and wrinkled, hanging in folds, and the scales are large, suggesting the skin of a lizard or aligator about their limbs or belly. His arms and legs remind one of the skin of the buffalo-perch, the carp, or other large fish. The cuticle every where is dry and harsh, and never perspires. There seems to be an absolute absence of fat, and the man is shrunken and withered, of a dead ashen-gray appearance, except here and there, where he is brown or brownish. Though only about fifty years of age, he impresses one as a very old man. The skin of the face is red and shining, and tightly drawn about the cheeks, pulling the lower lids down to such an extent as to perfectly evert them, making a horrid case of ectropion. In some places his scales are silvery, in others dark, and again in others are small and branny. His hair is very thin and dead-looking. The backs of his hands are fissured, and on his palms and soles the cuticle is greatly thickened. The fingers and toes seem shorter than natural, and the skin is drawn tightly back over both feet and hands. The septum between the fingers and toes seems to extend much farther down than usual, thus suggesting the webbed appearance before alluded to. He is considerably over six feet in height, and is a man of a low order of intelligence. He is married and is the father of severel children, none of whom, fortunately, inherit his malady, and as ichtyosis is almost if not always a congenital disease, they are not likely ever to have it. The fish-man fails to present but a single variety of icthyosis, and that is the porcupine disease, as it is called. In this spines, formed by hardened sebaceous material, protrude from the skin, closely packed together. Wilson states that he has observed them a quarter of an inch long. Willan reports having encountered them of an inch in length. I have never seen them longer than an eighth of an inch. Many years ago two brothers in England having this form of icthyosis were exhibited in the shows as porcupine-men.

Icthyosis is one of the rarest of skin-diseases. I am under the impression that it is more frequent in Europe than in this country. In ten years I have seen less than a dozen cases. Its cause, as I stated in my report to the American Dermatological Association, in 1877, is scrofula, according to my observation and experience. It is found in all the walks of life. I have encountered it with equal frequency among the rich and the poor. It is commonly considered incurable, and only temporarily and partially mitigable.

The treatment which I have found successful in permanently removing icthyosis, in more than one case, consists in the use of the constructives, *i. e.* cod-liver oil, extract of malt, syrup of the iodide of iron, syrup of the hypophosphites, etc.; attention to the digestive organs, and by giving the richest and best fat-producing foods, such as cream, butter, hog meat, fresh or cured, sugar and other sweets. A careful and thorough daily anointing with some oleaginous substance is of great value, and prolonged vapor or hot water baths should be employed frequently.

In conclusion I would advise every medical man, who may have the opportunity, to see this "Tennessee man-fish." He is positively grand in his dermal deformity, and presents, probably, an unequalled example of the fish-skin disease.—*Louisville Medical News.*

